# First Clinical Case of Ilizarov Femur Lengthening over a Bioactive and Degradable Intramedullary Implant

**DOI:** 10.1155/2023/7547590

**Published:** 2023-12-07

**Authors:** Arnold Popkov, Sergei Tverdokhlebov, Sergei Muradisinov, Dmitry Popkov

**Affiliations:** ^1^Russian Ilizarov Scientific Center for Restorative Traumatology and Orthopaedics, Kurgan, Russia; ^2^Tomsk Polytechnic University, Tomsk, Russia

## Abstract

**Introduction:**

The Ilizarov distraction osteogenesis is a recognized method of limb lengthening in orthopaedic practice. Its most challenging problems are long duration of external fixation and related pin-tract infection and joint contractures. The solution might be the use of a bioactive degradable intramedullary implant stimulating bone healing. *Case Presentation*. We present a case of a 14-year-old boy with 6 cm posttraumatic shortening of the femur and associated varus deformity of 20 degrees. He was treated with the Ilizarov technique of femur lengthening over an intramedullary degradable polycaprolactone (PCL) implant with hydroxyapatite (HA) filling. We faced no complications within the lengthening process. Shortening and deformity of the femur were corrected in 90 days. The index of external fixation was 15 days/cm. External fixation time was reduced almost twice comparing to the conventional method. Degradable intramedullary nails ensured the advantage of avoidance of the removal procedure. Radiography and CT confirmed faster new bone healing and remodeling.

**Conclusion:**

The combined lengthening technique over a PCL/HA implant might be used to shorten external fixation time and to stimulate bone healing especially in patients with compromised bone. Using a bioabsorbable material presents the benefit of eliminating the need for a second surgery to remove the nail, thereby reducing soft tissue damage.

## 1. Introduction

The Ilizarov method of bone reconstruction based on distraction osteogenesis is a unique method of bone tissue bioengineering due to its ability to generate vascularized bone tissue in vivo [1–3]. The evolution of distraction osteogenesis resulted in a number of technologies for the correction of upper and lower limb shortening of various etiologies, bone defects, and deformities [3–8]. The Ilizarov method is a recognized method for bone lengthening providing satisfactory results in the treatment of systemic diseases of the skeleton, congenital and posttraumatic bone discrepancy, and osteomyelitis [2–5]. Paying tribute to the advantages of the Ilizarov apparatus, long duration of external fixation (EF) that increases the likelihood of soft-tissue inflammation in the wire or half-pin tracts and discomfort for the patient still remain unresolved problems for orthopaedic surgeons applying the method [4–6].

As reported, the mean EF index ranges from 0.7 to 5.9 months/cm and depends on age, etiology, affected bone segment, and lengthening magnitude [6–9]. Therefore, many researchers and surgeons search for the ways to reduce the EF period by accelerating the rate of distraction, stimulating bone formation, and combining EF with intramedullary nailing [4–6, 10, 11]. It is well known that reparative bone regeneration depends on the osteogenic potential of the bone marrow [1]. However, thick intramedullary nails destroy it [12]. Moreover, one more operation is necessary to remove them if they fail mechanically or at patient's request. One of the modifications is the technology that combines EF and flexible intramedullary hydroxyapatite-coated nails [13]. According to the experimental studies conducted at our institution, it does not contradict the principles of the Ilizarov method and provides medullary blood supply as the bone marrow remains preserved [14]. The duration of EF with this technology required 20–33% fewer days than with the conventional Ilizarov technique. The elastic implants were left in situ or removed upon complete bone remodeling.

We continue searching for the ways of stimulating reparative regeneration in order to reduce EF wearing time. The solution might be the use of a suitable bioactive degradable intramedullary implant. To illustrate the use of this implant, a case is presented in which a femur lengthening was performed with the Ilizarov frame in combination with the biodegradable intramedullary implant.

### 1.1. Patient Information

**Figure 1:**
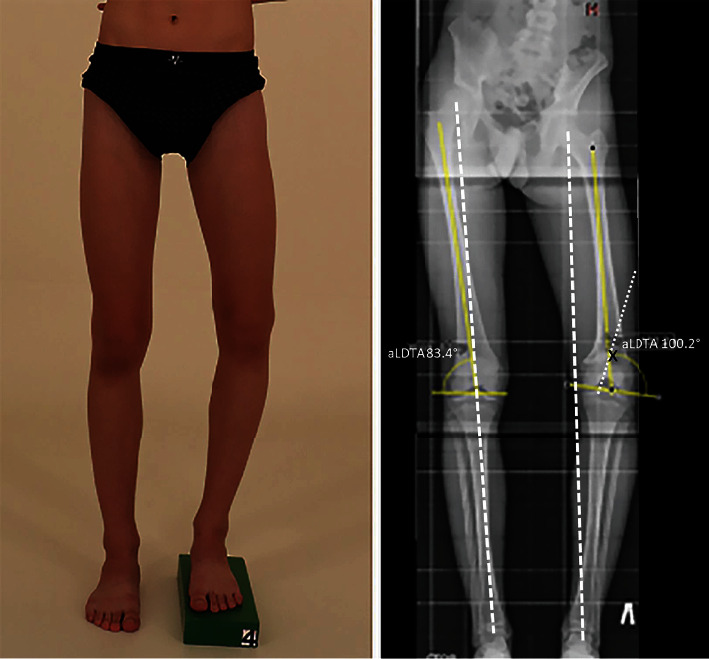
Preoperative photo and standing full-size radiograph of lower limbs: MAD (mechanical axis deviation) on the right side (0 mm) and on the left side (33 mm) of medial deviation; left side aLDFA (anatomical lateral distal femoral angle) 100.2°. Apex of the deformity (X) is situated at distal femoral metaphysis.

Our report presents a case of a boy with 6 cm posttraumatic shortening of the left femur and varus deformity in the distal femoral metaphysis of 20 degrees (Figure 1). He sustained an injury at the age of 10 while playing football. As there were no obvious signs of a fracture, it can be assumed that partial epiphysiodesis of the distal femur physis occurred after the injury. The parents gradually noticed the shortening and angular deformity and brought him to our institution for consultation at age 14.

At our institution, the patient and his family were proposed a new technology of limb lengthening over an intramedullary degradable polycaprolactone (PCL) implant impregnated with hydroxyapatite (HA). The implant materials were *ε*-polycaprolactone (Sigma-Aldrich, USA; Mn 80000) and hydroxyapatite (Fluidinova, Portugal; 10 ± 5 *μ*m). PCL was dissolved in high-purity acetone with a concentration of 15 wt %. Hydroxyapatite was preground in a ball mill in a ceramic chamber with ceramic grinding media with added acetone in a mass ratio of 1.5 : 1 at a rotation speed of 72 rpm for 12 hours. The PCL solution was added and mixed with HA in the ball mill. The mixture was poured in a thin layer into a preheated fluoroplastic mold. After drying, the composite was crushed in a low-speed polymer crusher (Shini SG-1621 N, Taiwan). Filabot EX2 single screw extruder (Filabot, USA) was used to obtain 4 mm wide filaments. Additionally, HA particles were applied to the implant surface by dipping into a suspension of HA powder in a solvent of known concentration and then dried to remove the residual solvent. The implants have mechanical properties: ultimate tensile strength 18.3 ± 2.4 MPa (by stretching) and 32.0 ± 3.4 MPa (by pulling) and elastic modulus 425.7 ± 21.9 MPa (by stretching) and 213.9 ± 8.8 MPa (by pulling). In comparison, the titanium alloy nails demonstrate ultimate tensile strength of 950 MPa (by stretching) and 1080 MPa (by pulling) and elastic modulus of 113.8 MPa (by stretching) and 110 MPa (by pulling) [14].

The parents signed an informed consent on the protocol of Ilizarov femur lengthening with a PCL/HA intramedullary nail. Ethics board permission was obtained.

### 1.2. Intervention

**Figure 2:**
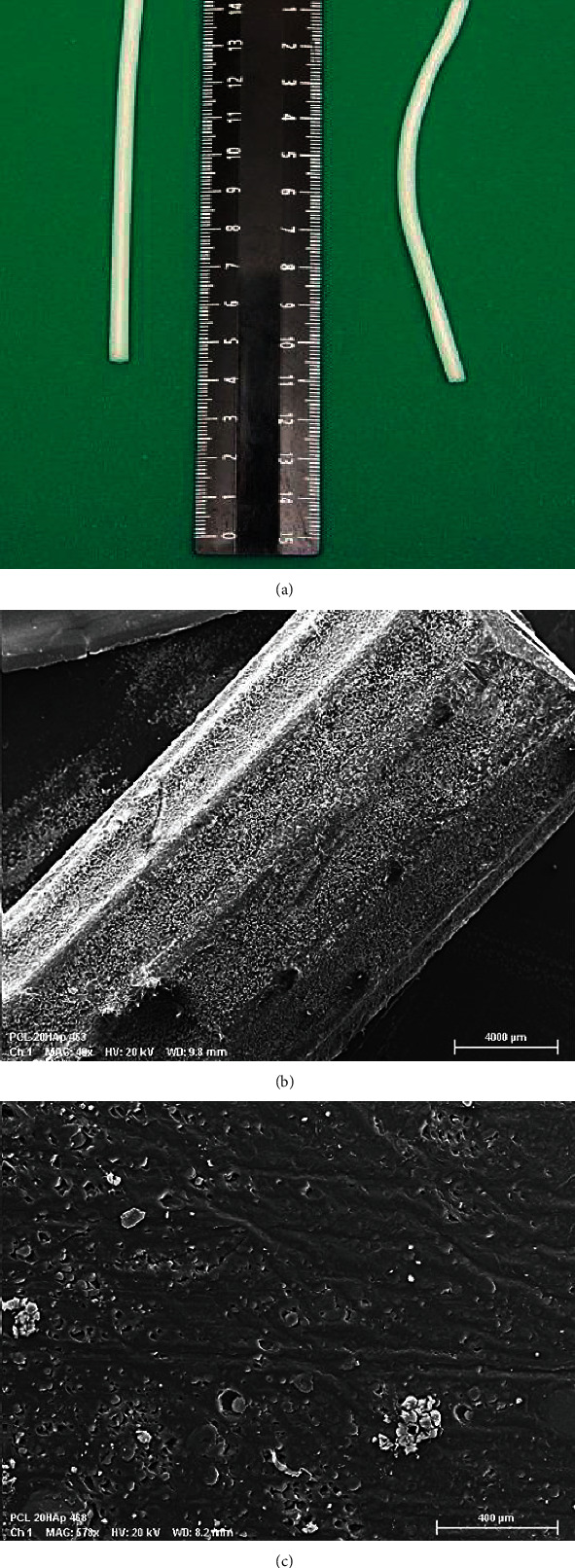
PLC/HA nails: (a) straight and bent nails; (b) nail's surface by electron scanning microscope (magnification: 49x); (c) transverse section of the implant; particles are evenly distributed throughout the depth of the polycaprolactone (magnification: 578x).

The intervention started with the introduction of a PCL/HA implant into the medullary canal. The implant was 100 mm long and 4 mm wide (Figures 2(b) and 2(c)). Approach was centered in front of lateral metaepiphysis. Through a skin incision of 3 cm and by soft-tissue dissection, a hole in cortex oriented forward medullary canal was formed in the distal femoral metaphysis (at a distance of 1 cm from the growth zone) with a 5 mm awl (the diameter is 1 mm superior to diameter of the implant). The use of awl ensured a straight tunnel for the implant in metaphyseal and distal diaphyseal part of the bone until to medullary canal. The slightly bent implant was inserted manually through this hole into metaphysis and then into the medullary canal. External part of the implant was cut, and soft tissues were sutured tightly.

**Figure 3:**
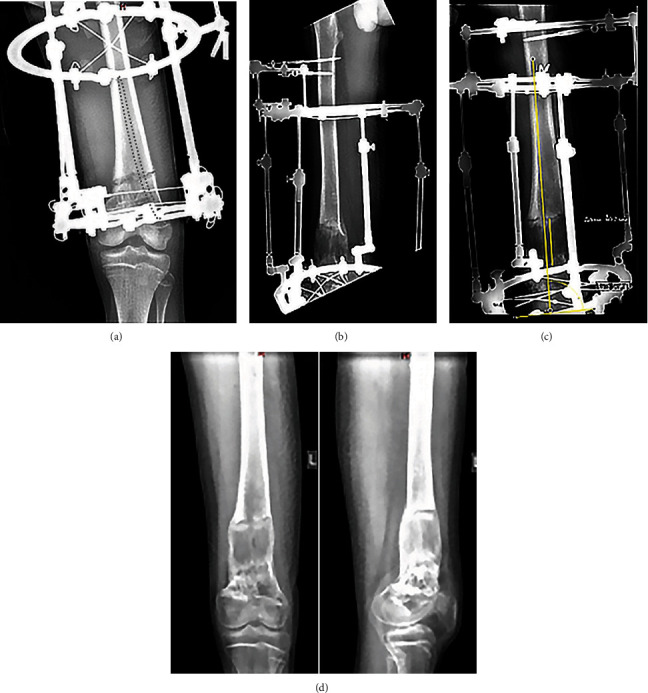
Radiographs of the left femur: (a) on the day of the intervention (position of the implant is not visible on radiographs; eventual position is presented with dashed grey line); (b) after 30 days of distraction; (c) at the end of distraction; (d) on the frame removal.

The next phase of surgery consisted of external frame application. The Ilizarov apparatus for lengthening of the distal femur with simultaneous correction of varus comprised three circular supports with two half-pins in the upper arch and five wires in the ring supports (Figure 3(a)). Planning of treatment included overlengthening on 5 mm and overcorrection of varus deformity of the left lower limb (mechanical axis deviation slightly lateral in comparison to contralateral right limb) because of existing medial part of distal femur physis. Partial corticotomy was performed with a conventional chisel and completed with osteoclasis. The wound was sutured, and aseptic dressings were applied. Upon radiographic control, the frame systems were stabilized. It is important to emphasize that the implant in the medullary canal does not interfere with the insertion of wires but requires strict implementation of the corticotomy technique. There is a risk of its cut if standard osteotomy is used, so none of the PCL/HA effects on osteogenesis in the regeneration gap might be expected.

### 1.3. Postoperative Management

Regular radiography for immediate and monthly bone regeneration control (Figure 3) was supplemented by CT upon completion of the lengthening protocol and the Ilizarov frame removal (Figure 3(d)).

**Figure 4:**
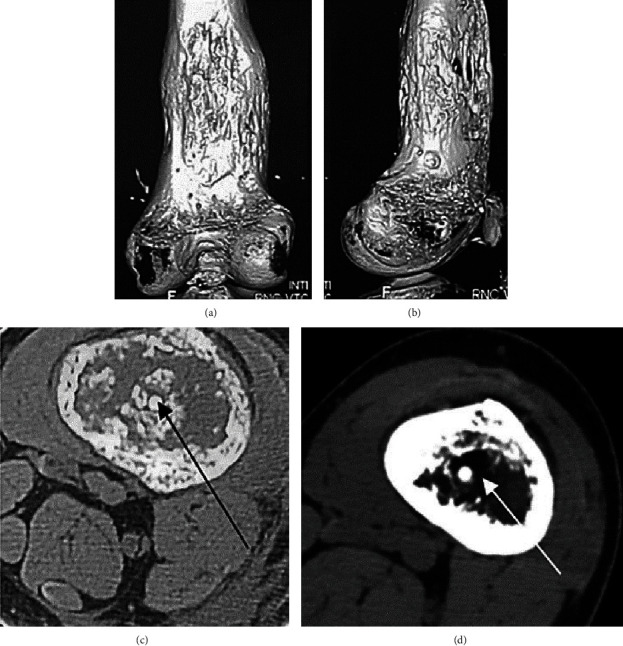
CT after frame removal: (a, b) 3D reconstruction of the bone regenerate in the distal femur upon frame removal; (c) transverse section of the distraction regenerate showing (arrow) exact position of the implant into newly formed bone at frame removal; (d) at 6-month follow-up.

There were no complications in postoperative period. Lengthening initiated 7 days after surgery at the rate of 1-1.5 mm daily divided into 4-6 events. Distraction phase lasted for 60 days. The radiographic shadow of bone regeneration filled the entire gap between the bone fragments after one month of distraction (Figure 3(b)). Unfortunately, the patient was found positive for COVID-19 with PCR test, and further lengthening continued first at the hospital for infectious diseases for two weeks and then on an outpatient basis (2,000 km from our clinic). Distraction continued for 60 days. Upon its completion, bone regeneration continuity was preserved and was most pronounced in the intermediary zone and along the center of the bone around the implant (Figure 3(c)). The optical density of the regenerate exceeded the optical density of the medullary canal of the proximal fragment. Another feature was a pronounced periosteal response (Figure 3(c)). Unfortunately, the epidemiological situation at the patient's place of residence allowed him to arrive at our clinic only a month after the end of the distraction in December 2021. The Ilizarov apparatus was removed after a clinical testing of bone healing (Figure 3(d)). CT confirmed a well-formed cortical layer and a well-mineralized central zone of the distraction regeneration (Figures 4(a)–4(c)). The overall EF continued for 90 days for 6 cm of lengthening amount. Thus, the EF index was 15 days/cm.

### 1.4. Clinical Outcome and Follow-Ups

**Figure 5:**
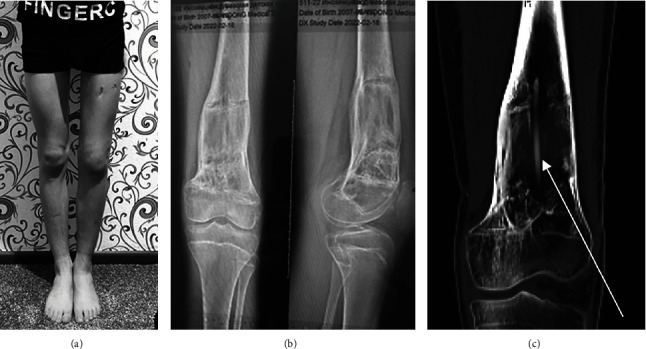
After frame removal: (a) photo; (b) AP and lateral view radiographs (2 months after frame removal); (c) CT longitudinal scan at 6-month follow-up with a PCL/HA implant (arrow) visible in newly formed bone.

The length of the legs was equal, and the limb was well aligned (Figure 5(a)).

The range of motion (ROM) in the hip and ankle was not limited, and active knee ROM was within 0°-0-30° degrees at frame removal. As the clinical test did not reveal any micromobility, the patient was recommended a gradual increase in weight-bearing. Two months later, the patient walked fully bearing weight on the involved limb and did not use any support. Knee ROM was 0°-0-60° degrees. CT at six-month follow-up showed a thickened regenerate and dense cortex (Figures 5(b) and 5(c)). The intramedullary canal continues formation (Figure 4(d)). The patient has followed a total of 9 months from index surgery and 6 months from EF removal.

## 2. Discussion

An external frame is a well-known and recognized method for bone lengthening [4, 15]. However, the classical Ilizarov femur lengthening of 6 cm needs two months of distraction and at least 4 months of fixation with the bulky Ilizarov frame on, followed by two-month rehabilitation to start full weight-bearing and walking without additional support [7–9, 15]. Therefore, our patient was proposed to trial a new technology that is aimed at reducing the total EF time. The case was posttraumatic, and it was supposed that lengthening might ran smoothly within the standard distraction protocol. It was hypothesized that the PCL/HA implant might promote bone remodeling in the fixation phase and after frame removal. Indeed, the study of patient's radiographs and CT images showed that the new technology provides optimal conditions for femur lengthening that may complete faster than reported by our and other studies that used the Ilizarov lengthening alone or in combination with intramedullary nailing [6–9, 15]. It was reported that the EF index of about 30 days/cm was considered an excellent result for femur lengthening, while 45 days/cm and 60 days/cm were judged as good and satisfactory ones [7–9]. In our case, it was twice shorter than the earlier excellent results. Moreover, the regenerate remodeling proceeded faster. It was accompanied by an expressed periosteal response and an increased cross-sectional area. We attribute the effects to the use of the PCL/HA implant.

Degradable implants have been intensively studied by researchers [16–22]. They should be fabricated from the materials that would be able to maintain the position of bone fragments until their consolidation and to decompose in the body under metabolism. Products from PCL or polylactic acid have been trialed as three-dimensional scaffolds to fill skull defects, in maxillofacial surgery, and as elastic matrices to replace damaged cartilage tissue [16, 17]. Screws and pins fabricated from such materials may reduce the weight of metal implants and the invasiveness of removing extraosseous or intraosseous implants. PCL is a degradable thermoplastic polymer used in a variety of medical applications, including bioprinting of tissues such as bone and cartilage due to its good biocompatibility, slow degradation rate, less acidic breakdown products in comparison to other polyesters, and strength for load bearing [17]. It is hydrolyzed into carbon dioxide and water through a metabolic process involving the citric acid cycle and excreted from the body.

Some currently used PCL implants are not bioactive [18, 19]. However, the investigation of absorbable implants has been aimed at enhancing their bioactivity [20–22]. The fundamental difference of the implant we offer is HA, both on its surface and body. Due to its proven osteoinduction properties, bone induction may begin from the first postoperative days involving the HA on the implant surface and continue actively as PCL starts decomposing releasing HA from the implant's deep layers. We observed a cancellous bone envelope around the implant that extended a few centimeters up the medullary canal from its proximal end. The CT showed that bone formation at the distal bone fragment significantly exceeded bone formation near the proximal one. It might be due to the fact that the distal implant end remained fixed in the metaphysis from the first days. Rapid formation of cortical plates and a pronounced periosteal reaction both on bone ends and at the level of the distraction gap were observed from the 10th day of distraction. At the end of distraction, the regeneration was optically continuous and did not feature a clear longitudinally oriented structure. We could explain this by the fact that bone trabeculae were formed not only under the effect of longitudinal tension stress but also around the implant, forming transverse bonds. Throughout the entire period of distraction, the enlightenment strip or “growth zone of the distraction regenerate”, typical for the classical Ilizarov distraction, was not so evident. A month after the end of distraction, mineralization of the regeneration around the implant was more intense in the distal femur (near the metaphysis) than in the proximal part. The period was characterized by a rapid increase in the optical density of the regenerate and by thickening of the continuous cortical plates. A well-formed cortical layer of the distraction regenerate, about 10 mm wide, and the central bone tissue around the implant were well identified, with the regenerate area of lower density between them. Regarding the time of full absorption of PCL/HA intramedullary nail, there is no evident data for the lengthening procedure. Bioabsorbable intramedullary nails developed for the management of paediatric diaphyseal forearm fractures were completely degraded in 57% of cases with at least four years of follow-up [18]. But the clinical and radiological outcomes in methods with degradable intramedullary implants can be assessed in a mean follow-up of 8.9 months [23].

Studies on the potential clinical use of PCL/beta-tricalcium phosphate implants for bone repair confirm our observation [24, 25]. To our knowledge, there are no works on the clinical use of PCL/HA implants for lengthening. We believe that the technology may be also relevant to congenital cases such as CPT, acquired bone defects, open fractures, and bone infection. The advantage of using HA implants is that they remain a depot of minerals for a long time. It is a special demand in the treatment of systemic skeletal diseases and osteoporosis. Owing to PCL decomposition, removal procedures are unnecessary. All these facts may result in financial benefits and have a psychological effect. We should emphasize the insignificant contribution of a PCL/HA nail for the mechanical stability of bone fragments in comparison to titanium elastic nails.

## 3. Discussion

Our case illustrates an effective use of a bioactive degradable PLC/HA implant in combination with the Ilizarov frame for femur lengthening. This lengthening technology might be beneficial from a clinical and social point of view as it decreases the external fixation index in comparison to the conventional Ilizarov technique and does not require another intervention for implant removal.

## Data Availability

The data supporting the results of the study can be sent upon simple demand to the corresponding author.
